# A Narrative Review on CD44’s Role in Glioblastoma Invasion, Proliferation, and Tumor Recurrence

**DOI:** 10.3390/cancers15194898

**Published:** 2023-10-09

**Authors:** Akihiro Inoue, Takanori Ohnishi, Masahiro Nishikawa, Yoshihiro Ohtsuka, Kosuke Kusakabe, Hajime Yano, Junya Tanaka, Takeharu Kunieda

**Affiliations:** 1 Department of Neurosurgery, Ehime University Graduate School of Medicine, 454 Shitsukawa, Toon 791-0295, Ehime, Japan; ma.nishikawa1985@gmail.com (M.N.); y.ohtsuka0818@gmail.com (Y.O.); kk_0145@yahoo.co.jp (K.K.); kuny@m.ehime-u.ac.jp (T.K.); 2 Department of Neurosurgery, Advanced Brain Disease Center, Washoukai Sadamoto Hospital, 1-6-1 Takehara, Matsuyama 790-0052, Ehime, Japan; 3 Department of Molecular and Cellular Physiology, Ehime University Graduate School of Medicene, 454 Shitsukawa, Toon 791-0295, Ehime, Japan; hajime-y@m.ehime-u.ac.jp (H.Y.); jtanaka@m.ehime-u.ac.jp (J.T.)

**Keywords:** glioblastoma, invasion, proliferation, CD44, tumor recurrence, hypoxia, phenotypic transition

## Abstract

**Simple Summary:**

As recurrence in glioblastoma is locally generated around the resection cavity, surviving glioma stem-like cells may cause recurrence. Glioma stem-like cells expressing high levels of CD44 are highly invasive and are required to change to less invasive, more proliferative types to generate a recurrent tumor. CD44 promotes both the invasion and proliferation of tumor cells via various signaling pathways. Among these, paired pathways show reciprocal activation of invasion and proliferation under different conditions of oxygenation. Severe hypoxia activates genes related to cell invasion, whereas moderate hypoxia activates genes related to cell proliferation. CD44 is associated with both pathways and plays a critical role in regulating the balance between the promotion of cell invasion and cell proliferation. These results may indicate that CD44 is a key molecule for executing tumor recurrence in glioblastoma.

**Abstract:**

High invasiveness is a characteristic of glioblastoma (GBM), making radical resection almost impossible, and thus, resulting in a tumor with inevitable recurrence. GBM recurrence may be caused by glioma stem-like cells (GSCs) that survive many kinds of therapy. GSCs with high expression levels of CD44 are highly invasive and resistant to radio-chemotherapy. CD44 is a multifunctional molecule that promotes the invasion and proliferation of tumor cells via various signaling pathways. Among these, paired pathways reciprocally activate invasion and proliferation under different hypoxic conditions. Severe hypoxia (0.5–2.5% O_2_) upregulates hypoxia-inducible factor (HIF)-1α, which then activates target genes, including CD44, TGF-β, and cMET, all of which are related to tumor migration and invasion. In contrast, moderate hypoxia (2.5–5% O_2_) upregulates HIF-2α, which activates target genes, such as vascular endothelial growth factor (VEGF)/VEGFR2, cMYC, and cyclin D1. All these genes are related to tumor proliferation. Oxygen environments around GBM can change before and after tumor resection. Before resection, the oxygen concentration at the tumor periphery is severely hypoxic. In the reparative stage after resection, the resection cavity shows moderate hypoxia. These observations suggest that upregulated CD44 under severe hypoxia may promote the migration and invasion of tumor cells. Conversely, when tumor resection leads to moderate hypoxia, upregulated HIF-2α activates HIF-2α target genes. The phenotypic transition regulated by CD44, leading to a dichotomy between invasion and proliferation according to hypoxic conditions, may play a crucial role in GBM recurrence.

## 1. Introduction

Glioblastoma (GBM) is the most common malignant brain tumor in adults and is characterized by a highly invasive nature and an extremely poor prognosis; the median survival is 15 months under the current standard treatment regimen based on the protocol outlined by Stupp et al. [1]. The poor prognosis may be largely attributable to early recurrence caused by tumor cells that escape tumor resection and develop resistance to radio-chemotherapy. Emerging evidence indicates that glioma stem-like cells (GSCs), which constitute a small subpopulation in GBM, may play a central role in GBM recurrence [2,3]. GSCs show stem cell properties of self-renewal and multi-lineage differentiation, and the ability to initiate a tumor on transplantation and recapitulation of the initial phenotype [4]. GSCs characteristically show highly invasive features and high resistance to radio-chemotherapy [5,6].

Solid tumors constitute tumor cells and a tumor microenvironment (TME). The TME in GBM includes astrocytes; neurons; endothelial cells; immune cells, such as non-tumor cells; extracellular matrix (ECM) components; and various growth factors and cytokines. In particular, the TME of cancer stem cells represents a niche in which cancer stem cells reside and mutually communicate with various components of the TME. Such bidirectional crosstalk between cancer stem cells and the TME can enable a wide variety of cellular events, such as invasion, proliferation, and angiogenesis [7,8].

In the TME, ECM molecules and their receptors play crucial roles in enacting this variety of cellular events. In the brain, the major component of the ECM is hyaluronic acid (HA) and its primary receptor is CD44. CD44 is a multi-functional transmembrane glycoprotein, and the binding of CD44 to HA can promote various tumor cell activities [9]. CD44 can mediate not only cell-to-cell interactions but also cell-to-ECM interactions, resulting in the transduction of microenvironmental signals to membrane-associated cytoskeletal proteins or the cell nucleus to activate the expression of various genes participating in cellular processes of migration, invasion, proliferation, and tumorigenesis in malignant tumors [10,11]. CD44 is also regarded as the most common stem cell surface marker in many cancers, such as those of the stomach, breast, lung, colon, and brain [12,13,14,15]. Thus, CD44 is not only an adhesion molecule but also a signal-transducing molecule that coordinately regulates numerous cellular behaviors through multiple signaling pathways.

GBM recurrences primarily occur at local sites of the marginal area around the resection cavity of the tumor [16,17]. Migrating GSCs with high invasive activity could be present in the peritumoral normal brain, escaping surgical resection and the following radio-chemotherapy and migrating further into the peritumoral area. Migrating GSCs that could cause tumor recurrence at the margins of the resection cavity would require at least two activities to accomplish recurrence. One is a homing ability to return to the original tumor site. The other is the ability to rapidly proliferate, i.e., to generate a tumor mass. According to the theoretical “Go or Grow” dichotomy of tumor cells [18,19], such behaviors of GSCs may require a phenotypic transition from invasive to proliferative tumor types. Herrlich et al. suggested that CD44 might display dual activities promoting the proliferation or invasiveness of tumor cells [20]. CD44 may thus play a crucial role in the regulation of invasion and proliferation as essential behaviors of tumor cells developing into GBM recurrence.

In this narrative review, we comprehensively review the literature that relates to the functional activities of CD44 and summarize recent advances in the activities of CD44. Using these data, we elucidate how CD44 can participate in the generation of tumor recurrence in GBM. A broad mapping of CD44 activities in the various cellular events, including invasion, proliferation, and epithelial-to-mesenchymal transition (EMT), and an understanding of how CD44-activated cellular events can be co-operatively regulated in GBM, particularly glioma stem-like cells, may bring new insight, not only clarifying the molecular mechanisms underlying GBM recurrence but also establishing a therapeutic strategy by effectively targeting CD44 to eradicate GBM.

## 2. Structure and Function of CD44

**Figure 1:**
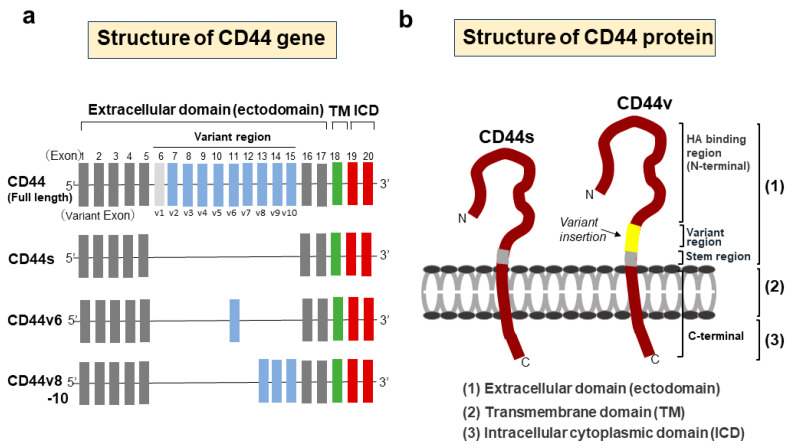
Structures of CD44 gene and protein. (**a**) Schematic structures of the CD44 gene. The CD44 gene encodes 20 exons, with exons 6–15 alternatively spliced and inserted into the stem region of CD44 as variant exons. The first five (1–5) and last five (16–20) exons are constant and encode the CD44 standard (CD44s) isoform. Splice variants with variant exons are designated CD44 variant (CD44v) isoforms. The structure of CD44v6 is shown as a representative variant isoform. In humans, v1 exon is lacking due to an in-frame termination codon. (**b**) Structural domains of the CD44 protein. CD44 consists of three domain regions, including an extracellular domain (ectodomain), transmembrane domain, and cytoplasmic domain. In CD44v, a variant region containing splice variants is inserted in the ectodomain. All CD44 isoforms have a hyaluronic acid (HA)-binding site in the extracellular domain.

The functional variety of CD44 is attributed to the diversity of molecular structures of CD44, including isoforms generated by the alternative splicing of the CD44 gene. The CD44 gene encodes 20 exons, with exons 6–15 alternatively spliced and inserted into the stem region of CD44 as variant exons (v1–v10) [21,22]. The first five (1–5) and last five (16–20) exons are constant and encode the shortest isoform of CD44, which is the CD44 standard (CD44s) isoform. Splice variants with variant exons are designated CD44 variant (CD44v) isoforms (Figure 1).

**Figure 2:**
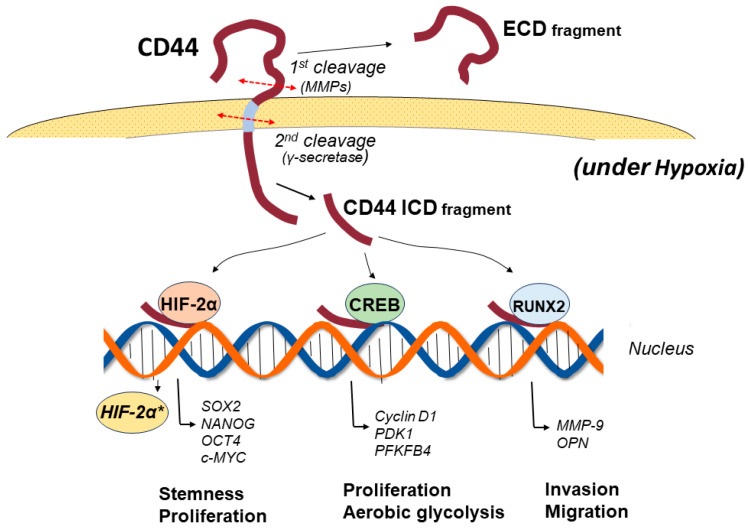
Signaling pathways of the CD44 intracellular domain (CD44-ICD) under hypoxia. CD44 is cleaved in two steps. The first cleavage is promoted via proteolysis of the ectodomain by matrix metalloproteinases, among others, followed by a second cleavage of the transmembrane domain by presenilin-dependent γ-secretase, resulting in the release of CD44-ICD. The released CD44-ICD fragments translocate to the nucleus and activate various genes as a co-transcription factor for Runt-related transcription factor 2 (RUNX2). CD44-ICD also binds to cAMP response element-binding protein (CREB) to regulate the gene expression of cyclin D1 and oxidative glycolysis-related genes 3-phosphoinositide-dependent kinase 1 (PDK1) and 6-phosphofructo-2-kinase/fructose-2,6-biphosphatase 4 (PFKFB4). Under hypoxic conditions, CD44-ICD binds to hypoxia-inducible factor (HIF)-2α and activates its expression, resulting in enhanced activation of HIF-2α target genes, including Sox2, NANOG, and OCT4, the genes of which are required for maintaining the stemness of GSCs. *: activated gene.

CD44 consists of three domains: an extracellular amino-terminal domain (ectodomain), a transmembrane domain, and an intracellular cytoplasmic domain (ICD) [23]. In CD44v isoforms, the stem region containing splice variants in the ectodomain provides a co-receptor for the binding of various growth factors and cytokines, thus transducing signals to promote invasion, progression, and tumorigenesis via specific signaling pathways [10]. Different isoforms of CD44 show different functions according to the variant exon inserted in the CD44 ectodomain. The amino-terminal globular region of the ectodomain is an HA-binding domain (HABD), where the HABD is common to all isoforms. HA binding to CD44-HABD causes conformational changes in CD44, resulting in the promotion of cell-signaling pathways to regulate a variety of cellular processes, such as migration and proliferation [24]. The transmembrane domain provides a pathway for interactions with co-factors, adaptor proteins, and receptor tyrosine kinases (RTKs) [25]. CD44-ICD is generated via intramembranous cleavage of CD44 via the proteolytic action of an enzyme complex formed by presenilin 1-presenilin 2-γ-secretase [26,27]. Proteolytic cleavage of CD44 is known to be sequentially performed in two steps [28]. Cleavage of the transmembrane domain of CD44 (second step) requires the preceding cleavage of the extracellular domain of CD44 (first step). The first step in the sequential cleavage of CD44 is achieved by two matrix metalloproteinases (MMPs), namely, a disintegrin and metalloproteinase (ADAM)10 and ADAM17, which are sequentially activated through the two separate pathways of elevated Ca2+ influx and activation of protein kinase C (PKC)-Rac, respectively [29]. The released CD44-ICD fragments then translocate to the nucleus and activate various genes as a co-transcription factor for Runt-related transcription factor 2 (RUNX2) [30]. Furthermore, CD44-ICD binds to the CCTGCG consensus sequence of CD44 (that is, the CD44-ICD response element (CIRE)) located near the RUNX2 site in the promoter of MMP-9, leading to upregulated MMP-9 expression. The CIRE sequence is also present in HIF-1α-related genes, the expression of which is regulated under normoxic conditions [31]. The activation of all these genes by CD44-ICD is achieved under normoxic conditions. In contrast, under hypoxic conditions, CD44-ICD binds to HIF-2α and activates its expression, resulting in the enhanced activation of HIF-2α target genes [32] (Figure 2).

The interactions of CD44 and HA can enhance, inhibit, or sometimes not show any effect on various cellular behaviors. CD44 was demonstrated to exist in three different activation states: inactive, inducibly active, and constitutively active [33]. Yang et al. reported that CD44 is inactive on normal cells, but active on cancerous cells [34,35]. The expression of the functional activities of CD44 and HA requires the activation of CD44 [36]. This activation can be induced by various factors, including growth factors, cytokines, and the TME [37]. In addition, although all CD44 molecules possess the HA-binding domain, affinities for HA binding differ. This suggests that variant isoforms of CD44 generated by alternative splicing in GSCs may participate in CD44 activation [38]. A more detailed understanding of the molecular mechanism underlying CD44 activation will no doubt provide new therapeutic strategies.

## 3. Expression of CD44 in GBM

Immunohistochemical investigations of CD44 and nestin in GBM demonstrated that CD44 is expressed on the cell membrane of GBM stem cells [39]. Si et al. recently reported that CD44 in GBM tissues is mainly expressed on the cell membrane, but some tumor cells express high levels of CD44 in the cytoplasm, with such high expression predicting a poor prognosis due to GBM [40]. We also reported that GBM expressing CD44 at much higher levels in the tumor periphery than in the tumor core shows much higher invasive features on magnetic resonance imaging (MRI), and patients presenting with high CD44 expression experience earlier recurrence and worse prognosis than those presenting with low CD44 expression [41]. Many studies reported that high expression of CD44 predicts poor prognosis in various cancers [42,43,44]. Immunohistochemical analysis of 125 breast cancer patients disclosed that the amount of CD44 protein correlates positively with poor disease-free survival and overall survival (OS) [45]. A meta-analysis of 583 patients with pancreatic cancer revealed that high levels of CD44 correlate adversely with the 5-year OS [46]. However, some studies reported no association between CD44 expression levels and survival. A meta-analysis of 1747 breast cancer patients showed no relationship between CD44 expression and OS [47]. Further, in GBM, high expression of CD44 is not significantly associated with low OS [48]. Although CD44 expression is recognized as a definite independent prognostic factor in various cancers, CD44v isoforms have increasingly attracted attention due to their much more specific expression in cancer stem cells and their various functions via signaling transduction compared with CD44. In esophageal squamous cell carcinoma, higher levels of CD44v9 protein at the invasive front of tumors correlate with worse progression-free survival (PFS) and OS [49], and CD44v9 independently predicts adverse prognosis in gastric cancer [50]. CD44v6 is reportedly predictive of metastasis for colon cancer [51] and has been associated with poor prognosis in several cancers, including prostate cancer and pancreatic cancer [52,53].

For GBM, few reports examined CD44v isoforms. To date, the expression of CD44v3, CD44v5, and CD44v6 isoforms have been identified in GBM, but their functions in terms of cellular processes remain unclarified, except for a role of CD44v6 in the regulation of GSC growth [54]. We investigated gene expressions of CD44s and CD44v in GSCs but found no significant difference in expression. GSCs obtained from the primary culture of tumor tissues in GBM expressing high CD44 showed that the expression of CD44v5 was the highest, followed by CD44v6, whereas differentiated GBM cells showed higher expression of CD44v6, followed by CD44v5. Stem-like cells obtained by introducing the Oct3/4 gene into differentiated glioma cells revealed the same expression pattern as GSCs (unpublished data). Wu et al. recently reported the results of a meta-analysis that evaluated the predictive value of higher CD44 expression for prognosis in patients with glioma [55]. That study disclosed that higher CD44 expression predicted poorer survival in patients with WHO grade II–III glioma but did not show any significant positive correlation with worse prognosis in patients with GBM. As potential reasons for this discrepancy, the authors suggested the molecular heterogeneity of GBM and the fact that the optimal cut-off values for CD44 expression had not been determined for each grade of tumor. We recently reported that patients with GBM showing high CD44 expression in the tumor periphery show significantly shorter survival in terms of both PFS and OS than those showing low CD44 expression [56]. In the future, a novel CD44v isoform that can more definitively predict prognosis in patients with GBM may be identified.

## 4. Signaling Pathways via CD44-HA Interactions

**Figure 3:**
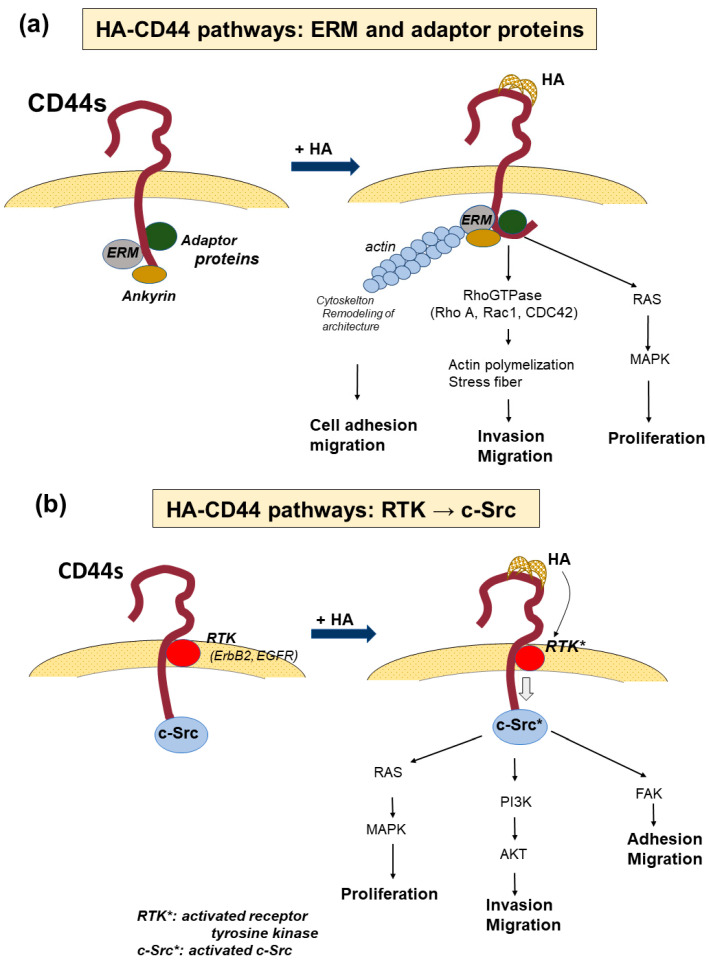
The signaling pathway activated by HA-CD44 interaction. (**a**) The signaling pathway from the promotion of the binding of ezerin-radexin-moesin (ERM), ankyrin, and adaptor proteins to the cytoplasmic tail. Binding activates the interaction of CD44 and RAS and RhoGTPase, leading to the promotion of actin polymerization, remodeling of the cytoskeletal architectures, and facilitating cell migration and invasion. In contrast, the oncogenic gene RAS activates mitogen-activated protein kinase (MAPK) and promotes cell proliferation. (**b**) Signaling pathway activated by receptor tyrosine kinases (RTKs). HA-CD44 interaction activates RTKs, such as ErbB2 and EGFR, which elevate the activities of the non-receptor kinases of the Src family or Ras family GTPase. Such intracellular signaling enhances the activity of downstream signaling pathways, such as MAPK, phosphoinositide 3-kinase (PI3K)/AKT, and focal adhesion kinase (FAK). The former promotes cell proliferation, while the latter two enhance cell migration and invasion. *: activated gene.

CD44 binds to various ligands and the interactions allow cancer cells to execute many cellular functions via the various signal transductions specific to the ligands. Among these, common ligands for CD44 include HA, fibronectin [57], osteopontin (OPN) [58], collagen [59], and serglycin/sulfated proteoglycan [60]. The effects of CD44 on tumor cell invasion, proliferation, and progression depend on the specificity of the interactions between CD44 and each ligand. HA, which is a major constituent of the ECM, is the most common ligand of CD44 and its interactions with CD44 can activate multiple pathways, leading to cell survival, proliferation, and invasion via signaling networks, including pathways through adaptor molecules; RTKs, such as EGFR and ErB2; and non-receptor kinases of the Src family [10,61] (Figure 3a,b).

As CD44 lacks kinase activity, several signaling systems are utilized to activate protein kinases. The binding of HA to CD44 causes a conformational change in CD44 to facilitate binding to the ezrin-radoxin-moesin (ERM) protein and other adaptor molecules via the cytoplasmic tail of CD44, which mediates CD44 interactions with Ras and RhoGTPase, including RhoA, Rac1, and CDC42 as effectors of upstream pathways [62]. Activation of these molecules initiates changes in the cytoskeletal architecture to facilitate tumor cell migration and invasion into the extracellular space of the ECM [61]. HA-CD44 interactions also activate receptor tyrosine kinases, such as ErbB2 and EGFR, in the signaling domain of CD44 and elevate the activities of non-receptor kinases of the Src family or Ras family GTPases [63]. These intracellular signaling pathways enhance the activity of downstream signaling pathways, such as mitogen-activated protein kinases (MAPK), phosphoinositide 3-kinase (PI3K)/Akt, and focal adhesion kinase [63]. Through the Ras/MAPK pathway, Src signaling promotes tumor cell division and proliferation. In contrast, the PI3K/AKT pathway contributes to tumor cell invasion and proliferation [61,64]. In addition, HA binding to CD44 activates multidrug transporters that participate in chemoresistance [65].

**Figure 4:**
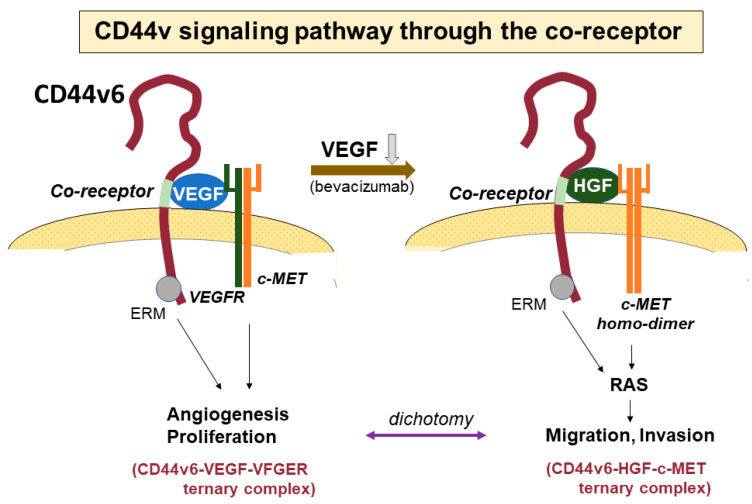
The signaling pathway through co-receptors of CD44. The CD44v6 isoform has a co-receptor in the variant region of the CD44 ectodomain. Vascular endothelial growth factor (VEGF) and hepatocyte growth factor (HGF) can bind to the co-receptor. VEGF bound to the co-receptor further binds to the VEGF receptor (VEGFR), forming a ternary complex of CD44v6-VEGF-VEGFR to promote angiogenesis. VEGFR and cMET form a heterodimer and suppress migratory activity via the interaction of HGF and cMET. In contrast, the binding of VEGF to CD44 is inhibited by the anti-VEGF antibody bevacizumab, and thus, CD44v6 does not form the heterodimer of VEGFR and cMET and a homodimer with cMET is instead generated. HGF then binds to the co-receptor of CD44 instead of VEGF, creating a CD44v6-HGF-cMET ternary complex. This stimulates the Ras signaling pathway, resulting in increased motility.

Specific exons at some sites in the ectodomain of CD44 are inserted via alternative splicing in CD44v isoforms, as mentioned above. These peptide sites in CD44v isoforms can act as co-receptors for various growth factors and receptors, resulting in participation in specific signaling pathways (Figure 4). In the CD44v3 isoform containing a heparin sulfate site, heparin sulfate-binding growth factors, such as fibroblast growth factor (FGF) and epidermal growth factor (EGF), bind to CD44 [24]. CD44v6 has a binding site for hepatocyte growth factor (HGF) and VEGF [25]. As HGF is a ligand for cMET, the interactions of these molecules activate cMET, leading to stimulation of the Ras signaling pathway and a subsequent increase in motility [66]. In the treatment of both primary and recurrent GBM, antiangiogenic therapy with bevacizumab, which is a monoclonal antibody that targets VEGF, is often performed. However, in patients that show resistance to bevacizumab, the tumor often presents much more invasive features than seen before treatment. McCarty reported that tumor cell proliferation and invasion are regulated via crosstalk between the VEGF and HGF signaling pathways [67]. When GBM proliferates and shows progression, VEGFR-2 and cMET form heterodimeric complexes that inhibit cell invasion via the tyrosine dephosphorylation of cMET. When VEGF is neutralized with bevacizumab, cMET is upregulated, forms homodimers that allow for the binding of HGF, and promotes mesenchymal-like phenotypes and tumor cell invasion. In our study, the inhibition of VEGF with siRNA or bevacizumab in GSCs also markedly increased the expressions of both CD44 and cMET and caused much higher tumor invasion than the control [68].

CD44-intracellular cytoplasmic domain (ICD) plays crucial roles in the intracellular signaling pathways for executing special cellular events, such as tumor progression, the maintenance of the stemness of cancer stem cells, aerobic glycolysis, and the production of CD44. As the structure and generation of CD44-ICD were described in the previous section, the major pathways of CD44-ICD are detailed here (Figure 2, Table 1). The expression of CD44-ICD is specific to cancer stem cells, and the hypoxic environment greatly affects CD44-ICD pathways. Under normoxic conditions, CD44-ICD activates HIF-1α-related genes without the binding of HIF-1α to CD44-ICD [32]. In contrast, under hypoxic conditions, CD44-ICD can bind to HIF-2α and activate the expression of HIF-2α. The enhanced activity of HIF-2α upregulates HIF-2α-target genes, such as SOX2, OCT4, NANOG, and c-MYC [32,69]. Hypoxia-induced HIF-2α is preferentially expressed in cancer stem cells compared with differentiated tumor cells [70] and plays an important role as a transcription factor to activate genes related to the maintenance of stem cell features. In addition, CD44-ICD activates the transcription of genes containing 12-O-tetradecanoyl-phorbol-13-acetate response elements, such as CD44 [71]. The cleavage of CD44 facilitates detachment from HA in the ECM. CD44-ICD liberated by the second cleavage step can elevate the expression of newly generated CD44 molecules on the cell membrane, facilitating binding to other sites and resulting in the promotion of cell migration and invasion. CD44-ICD also binds to the cyclic adenosine monophosphate (cAMP) response element-binding protein (CREB) to regulate the expression of genes, such as cyclin D1 [72] and the oxidative glycolysis-related genes 3-phsphoinositide-dependent kinase 1 (PDK1) and 6-phosphofructo-2-kinase/fructose-2,6-biphosphatase 4 (PFKFB4) [73]. Activation of these genes can promote the proliferation, aerobic glycolysis, and creation of new CD44 molecules in GSCs.

**Figure 5:**
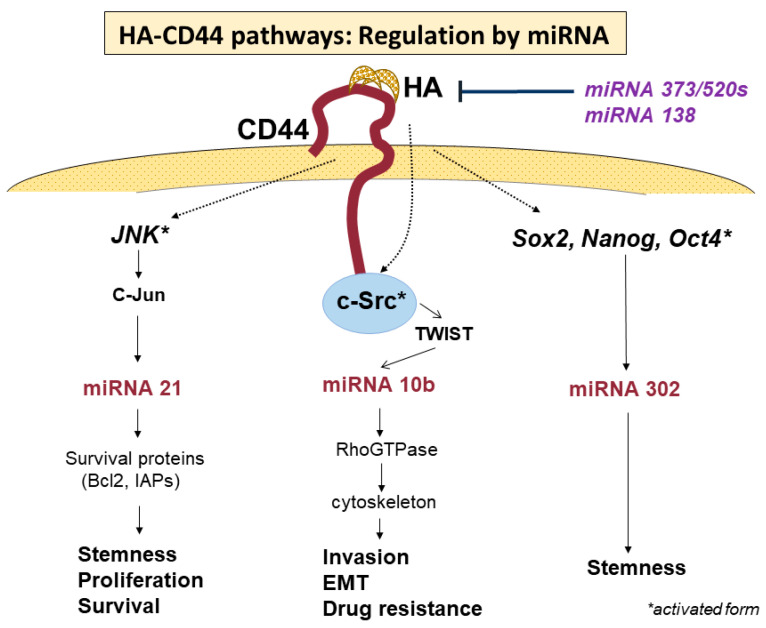
Regulation of HA-CD44 binding-activated signaling pathways. Activity of the signaling pathways promoted by HA-CD44 binding is regulated by miRNAs from both upstream and downstream. Each miRNA regulates the signaling pathway to promote special cellular behaviors.

As mentioned above, HA-binding CD44 activates numerous signaling pathways that participate in various cellular events of cancers, including migration, invasion, proliferation, survival, stemness, drug resistance, and many other functions involved in malignant properties. However, the question remains as to how cancer cells can regulate the numerous signaling pathways in a coordinated manner, leading to the tumor-specific phenotypes and behaviors seen during cancer development and progression. Bourguignon et al. demonstrated that HA-CD44 interaction also activates microRNA (miRNA) signaling to regulate tumor-specific functions, such as tumor cell growth, migration, invasion, and chemoresistance, thus promoting tumor progression [74] (Figure 5, Table 2). As small, non-coding RNAs of approximately 22 nucleotides in length, miRNAs mediate the posttranscriptional silencing of specific target mRNAs and are recognized as important conductors that regulate tumor development and progression [75]. Many miRNAs were reported to show aberrant expression in various cancers. In this review, we describe primary miRNAs expressed in GBM and their pivotal roles in tumor-specific malignant behaviors. Oncogenic miRNA-21 is known to regulate tumor cell proliferation, invasion, survival, chemoresistance, and progression in various cancers [76,77,78]. HA-binding CD44 interactions were demonstrated to activate miRNA-21 via the phosphorylation of the JNK/c-Jun transcription factor [79]. Chen et al. found that the inhibition of JNK/c-Jun or miRNA-21 downregulated the expressions of survival proteins, such as Bcl2 and inhibitors of apoptosis protein (IAPs), resulting in increased apoptotic cell death and the decreased chemoresistance of tumor cells [80]. These findings suggest that miRNA-21 upregulated by JNK/c-Jun, which is activated by HA-CD44 interactions, may play a crucial role in tumorigenesis and chemoresistance. Several studies reported that miRNA-21 is significantly elevated in GBM. Gabriely et al. reported that the inhibition of miRNA-21 by antisense oligonucleotides leads to elevated levels of RECK and tissue inhibitor of metalloproteinase (TIMP) 3, thus reducing MMP activities, both in vitro and in vivo. In addition, the downregulation of miRNA-21 in glioma cells decreased their migratory and invasive abilities [81]. Luo et al. found that miRNA-21 promotes the migration and invasion of glioma cells by activating Sox2 and β-catenin signaling [82]. Further, miRNA-21 was reported to participate in the drug resistance of GBM [83].

In addition to miRNA-21, the overexpression of miRNA-10b was found to be necessary to promote the migration and invasion of tumor cells in the metastatic process of breast cancer cells [84]. Sasayama et al. reported that miRNA-10b is overexpressed in malignant gliomas, being the highest in GBM, and that the overexpression of miRNA-10b is associated with the upregulation of Rho C, which stimulates glioma invasion and migration [61,85]. On the other hand, miRNA-10b is reportedly activated by phosphorylated TWIST via the activation of c-Src under the stimulation of HA-CD44 interaction, promoting tumor-cell-specific activities, such as EMT, tumor invasion, and drug resistance, in cancer cells [86]. Furthermore, miRNA-302 was demonstrated to promote the stemness of cancer stem cells [87,88,89], which is upregulated by transcription factors such as Nanog, Oct4, and Sox2 [89,90,91], the expressions of which are activated by HA-CD44 interaction [92]. The treatment of cancer stem cells with anti-miRNA-302 inhibitors upregulates lysine-specific histone demethylases and decreases DNA global demethylation, impairing HA-CD44-activated functions in cancer stem cells [93]. These results indicate that the signaling pathway of miRNA-302 may be regulated by stem cell markers, such as Nanog/Oct4/Sox2, for which the expressions are activated by HA-CD44 interactions [93].

**Table 2:** microRNAs participating in regulation of the activites of CD44 in glioblastoma.

microRNA	Regulation	Activated or Inhibitted Cellular Function of CD44	References
miR-21	positive	Activation: Proliferation, stemness, invasion, chemotherapy resistant	[76,77,78,80,81,82]
		Inhibition: senescence, apoptosis	[83]
miR-10b	positive	Activation: Invasion, migration, EMT, drug resistance	[61,84,85,86,93]
miR-302	positive	Activation: stemness	[87,88,89,90,92]
miR-373	negative	Inhibition: proliferation, invasion	[94]
miR-520s			
miR-138	negative	Inhibition: proliferation, cell cycle, migration, wound-healing ability	[95]

EMT; epithelial-to-mesenchymal transition.

On the other hand, some miRNAs are known to regulate the expression and functional activities of CD44. Feng et al. demonstrated that CD44 is a direct target of miRNA-373 and miRNA-520s, and the expressions of miRNA-373 and miRNA-520s are negatively associated with CD44 expression in GBM [94]. Suppressing CD44 expression via the increased expressions of miRNA-373 and miRNA-520s leads to the inhibition of the proliferation and invasiveness of GBM cells [94]. Yeh et al. reported that whole-transcriptome analysis and miRNA expression profiling identified miRNA-138 as one of the most significantly downregulated miRNAs [95]. Downregulated miRNA-138 shows an inverse correlation with CD44 expression. An overexpression of miRNA-138 in GBM cells reduces CD44 expression and inhibits cell proliferation, cell cycle, migration, and wound-healing capabilities. They revealed that miRNA-138 negatively regulates the expression of CD44 by directly binding to the 3′-untranslated region (UTR) of CD44. Thus, CD44 inhibition by miRNA-138 results in an inhibition of GBM cell proliferation via cell cycle arrest by inducing p27 and subsequent translocation of this molecule into the nucleus. Such suppressive activities of miRNAs may have much more beneficial effects as therapeutic targets than anti-CD44 therapy targeting each signaling pathway. As mentioned above, CD44 signaling pathways are regulated in a very complicated manner by transcriptional factors, protein kinases, miRNAs, and so on. Among these, miRNAs may play primary roles in regulating the activities of CD44 related to cellular events. However, the detailed mechanisms remain unclear. The elucidation of these mechanisms underlying the regulation of CD44 signaling pathways will be required to develop novel therapeutic strategies.

## 5. CD44-Promoted Tumor Invasion in GSCs

CD44 is well known to promote tumor migration and invasion in GBM [96]. The mechanisms of glioma invasion are increasingly being understood via in vitro and in vivo models, but the results have yet to be translated into clinical applications. GBM cells, particularly GSCs, were shown to be more migratory and invasive than their non-GSC counterparts [97]. GSCs are highly migratory and invade normal brain parenchyma around the tumor mass in a coordinated manner, allowing for escape from surgical resection and adjuvant therapies and providing the major source of tumor recurrences. The invasive ability of tumor cells is determined by interactions between tumor cells and the ECM, as well as non-tumor cells, such as endothelial cells, astrocytes, and neurons. The ECM plays important roles, not only in providing a scaffold for tumor cells to adhere to and move along but also in intra- and inter-cellularly transducing signals. These interactions in GSCs are generally regulated by hypoxic conditions as special microenvironments called niches. Hypoxia enhances the invasiveness of GBMs with links to the increased production of HA [98]. In addition, higher levels of HA are expressed at the invasive edge of a GBM [99]. We found that severe hypoxia (1% O_2_) increases the expression of CD44 via the activation of HIF-1α expression, resulting in enhanced GSC migration and invasion. Enhancements in both CD44 expression and GSC invasion were significantly decreased by silencing the HIF-1α gene with its siRNA [100].

Cell migration and invasion are highly integrated, multistep processes that can be accomplished via the interactions of tumor cells and various components in the TME, particularly the ECM. These processes include the adhesion of tumor cells to adjacent cells, such as endothelial cells, neurons, astrocytes, and the ECM; changes in tumor cell morphology via reorganization of the intracellular cytoskeleton so that the tumor cell can more readily pass through the narrow spaces of the ECM; and diffuse migration into the normal brain tissues around the tumor mass with remodeling of the ECM via proteolytic degradation using matrix metalloproteinases, such as MMP-2 and MMP-9, to promote detachment from the adhered ECM and movement to a new site of adhesion [101,102]. The interaction of CD44 and HA activates PI3K, by which phosphatidylinositol diphosphate (PIP2) is converted to PIP3. This PIP3 then activates Rho GTPases, including Rho A, Rac 1, and Cdc42. Rho GTPase signaling plays critical roles in cell motility and the invasion of GBM cells via the polymerization of actin to produce stress fibers, filopodia, and lamellipodia [62].

GSCs expressing high levels of CD44 thus bind to HA, and the amounts of HA are increased via excretion from GBM cells, promoting cell migration and invasion via the signaling pathways of Rho GTPases and PI3K. On the other hand, integrins, which are another type of major cell adhesion receptor comprising α and β transmembrane glycoproteins [103], also stimulate tumor cell migration and invasion in GBMs via similar signaling pathways, including focal adhesion kinase, PIP3K, and AKT kinases [104]. An important question concerns how tumor cells use the two different signaling pathways of HA binding to CD44 or integrins binding to their ligands. HA is a major component of the ECM in the brain parenchyma, while the ligand molecules for integrins, such as collagen, fibronectin, and laminin, are generally lacking in the brain parenchyma. Accordingly, the major signaling pathway to elevate tumor cell motility and promote diffuse invasion into the brain parenchyma around the tumor mass in GBMs is thought to involve the interactions of HA and CD44 binding. In contrast, the interactions of integrins and their ligands are likely to have a critical function in the migration and invasion of tumor cells along the perivascular space and white matter tracts in GBM [105,106]. Binder et al. reported that the migration and invasion of GBM cells are unaffected by the knockdown of integrin α (ITGAV), but are decreased by the knockdown of CD44 using a mouse organotypic slice model [107]. Wolf et al. demonstrated a mechanism by which tumor cells adhere and migrate through the nano-porous, three-dimensional ECM of brain tissue [108]. As the mechanism, GBM cells are likely to adhere to and invade HA-rich ECM using microtentacles that extend tens of micrometers from the cell body via binding to the CD44 receptor. The study also provides insights into how CD44 supports cell motility. CD44 is sufficient to drive the formation of tension-bearing protrusions that can activate cell motility and invasion without the participation of integrins. They also previously reported that CD44-HA-promoted adhesion occurs more rapidly but is weaker than integrin-activated adhesion [109]. These data suggest that CD44-HA binding may be characterized by many rapid, weak interactions with the ECM, whereas integrin binding is characterized by fewer, slower interactions but with an ability to reinforce CD44-HA interactions.

Recently, the Ca^2+^ signaling pathway and ionotropic glutamate receptors were reported to affect the motility and invasiveness of GBM cells [110]. As described above, glioma cells shape the TME to promote their migration and invasion by modifying and degrading the ECM.

## 6. CD44-Promoted Tumor Proliferation in GBM (GSCs)

Although CD44 is a multi-functional receptor molecule that promotes invasion, proliferation, drug resistance, angiogenesis, tumorigenesis, and EMT in malignant tumor cells, the signaling pathways for these cellular events are thought to be activated according to the requirements of tumor cells to display each cellular event under pathological conditions. On the other hand, tumor cells were noted to show the dichotomy of either cell proliferation or migration, representing the “Go or Grow” theory [18]. That is, when tumor cells move, tumor cells do not grow, and vice versa [19,111]. How CD44 participates in the cellular processes of this dichotomy in the invasion and proliferation of GBM cells remains unclear. The possible mechanisms by which CD44 primarily promotes tumor cell proliferation with suppression of tumor cell invasion need to be determined. We describe the signaling pathways and their regulation in which CD44 transduces intracellular and extracellular molecules to promote tumor proliferation. Xu et al. demonstrated that CD44 promotes the proliferation of GBM cells, both in vitro and in vivo, by attenuating the activation of the Hippo signaling pathway as a single downstream pathway of CD44 [112]. This result supported a previous report that merlin, functioning upstream of the Hippo signaling pathway, binds to CD44 and inhibits GBM growth by interfering in the interaction of CD44 and HA [113]. Jijiwa et al. reported that the knockdown of the CD44v6 isoform with its siRNA inhibits in vitro growth in brain tumor (GBM) stem cells with high expression of CD44v6 cells, but not in GBM stem cells with low expression of CD44v6. Also, the overexpression of mouse CD44v6 or OPN enhances the growth of mouse stem-like tumor cells, and this growth may be dependent on the CD44/AKT pathway [54]. We also found that OPN upregulated by HIF-2α under 5% O_2_ (moderate hypoxia) enhances the proliferation of GSCs and that silencing the OPN gene with its siRNA inhibits the cell growth, but elevates the expression of CD44, leading to the promotion of tumor cell migration and invasion [100]. Pietras et al. reported that OPN-CD44 interactions not only enhance cancer stem cell phenotypes but also promote aggressive tumor growth in vivo in GSCs existing in the perivascular niche of proneural GBM [15]. They also demonstrated that these cellular activities are due to the activation of CD44-ICD that has been released into the cytoplasm via the cleavage of CD44 by γ-secretase, which, in turn, is activated by the interaction between OPN and CD44. As described above, CD44-ICD is an intracellular free fragment that translocates to the nucleus and activates the transcription factors of various genes, resulting in enhanced tumor proliferation, glycolytic metabolism, and increased stemness in tumors (particularly as cancer stem cells). Under moderately hypoxic conditions, CD44-ICD binds to HIF-2α and upregulates the expression of HIF target genes. De Falco et al. reported that CD44-ICD enhances the expression of cyclin D1 by promoting CREB recruitment to the cyclin D1 promoter and upregulating the transcription of cyclin D1, leading to the acceleration of cell proliferation in thyroid carcinoma [72,114].

Daniel et al. investigated the intratumoral activities of the MAPK and PI3K pathways in GBM, demonstrating a striking preponderance toward the mutual exclusivity of MAPK and PI3K activations in GBM tissue [115]. That is, MAPK activation correlates with proliferation and transcription factor CREB activation in the region of the tumor proliferation area, whereas PI3K activation correlates with CD44 expression, which strongly promotes tumor invasion in the region of the tumor border zone in GBM. Daniel et al. also showed that MAPK-active regions correlate with the expression of proliferating cell nuclear antigen (PCNA), MMP-9, and pCREB (a pro-oncogenic transcription factor that regulates genes related to proliferation) [114], but with little or no expression of CD44. In contrast, PI3K active regions exhibit high degrees of CD44 expression but little or no expressions of PCNA, MMP-9, or pCREB. These studies identified intratumoral heterogeneity in the activities between invasion and proliferation. The mutually exclusive expression of these activities may relate to a phenotypic transition that may be an essential behavior of tumor cells to develop local recurrence or distant metastasis in cancer cells. Consequently, a better understanding of the role of phenotype in tumor cells showing different activities of CD44 is required to create therapeutic strategies that target CD44 in GBM.

The activity of multiple functions promoted by CD44 is regulated by various factors, as described above. Among these, suppressive miRNAs, such as miRNA-138 (which can not only suppress the proliferation of GBM but also downregulate whole activities of CD44), may be potential targets for the establishment of effective therapies against GBM. While miRNAs may be effective at inhibiting tumor cell proliferation, examination is required to clarify whether such miRNA-regulated inhibition of proliferation affects the invasive activity in GBM cells.

## 7. Roles of CD44 in Phenotypic Transition in GBM Progression

GBM exhibits a high degree of inter- and intra-tumoral heterogeneity, reducing the anti-tumor effects of radiotherapy and chemotherapy in the treatment of both primary and recurrent GBM. Heterogeneity is confirmed to occur at various levels, including the histopathological, transcriptional, and genomic stages [116,117,118]. The heterogeneity, particularly intra-tumoral heterogeneity, is believed to be largely due to the phenotypic transition of GBM to a mesenchymal (MES) subtype [119]. The Cancer Genomic Atlas (TCGA) identified four molecular subtypes of GBM, which were associated with prognostic values via a large-scale genomic analysis of GBM, including proneural (PN), neural (NL), classical (CL), and MES types [120,121]. In 2017, among those four types, TCGA defined three subtypes, namely, PN, CL, and MES subtypes, in which the intrinsic genetic signature of each subtype correlated with the tumor immune microenvironment [122]. In the study, Wang et al. selected patients with GBM without the IDH mutation and established a relationship between treatment and phenotypic transition in recurrent tumors of GBM. By comparing 91 recurrent tumors, they found that the PN subtype showed the highest transition rate in which 59% of patients with the PN subtype shifted to MES or CL types, while 33% of patients with the MES subtype shifted to other subtypes [122]. These subtypes show different prognoses, with the worst in the MES subtype [122]. Over the course of tumor progression, GBM often shifts in subtype from the original to other types, and the most prominent shift was found in the PN-to-MES transition (PMT) [123]. The PMT is synonymous with the EMT in cancer cells of epithelial origin [124]. These MES-type tumors show increased motility, invasiveness, and metastatic potential, in addition to greater resistance to radio- and chemotherapies [125]. However, to develop a tumor mass at the metastatic site, the tumor is required to reproduce the original epithelial features via a reverse transition from an MES to an epithelial phenotype (MET) because the epithelial phenotype has features better suited to aggressively proliferating tumor cells compared with the MES phenotype [126]. Also, in the process of tumorigenesis on recurrence in GBM, highly migratory and invasive GSCs, both primarily and secondarily after radio-chemotherapy, are expected to show a change in phenotype to a less invasive, more proliferative phenotype (epithelial-like type) to reproduce the original tumor mass. Accordingly, an understanding of the mechanisms underlying the EMT in GBM cells may give clues to the elucidation of the molecular mechanisms underlying tumor generation on GBM recurrence.

Extensive studies revealed that high CD44 expression correlates with a mesenchymal phenotype of cancer stem cells and participates in the EMT, resulting in tumors with worse prognosis showing highly invasive, metastatic, and chemo-radiation-resistant features [125,127]. The EMT represents the adaptive plasticity of cancer cells [128], a cell behavior by which tumor cells change phenotype in response to a microenvironment where favorable conditions are provided to tumor cells to enhance invasiveness, proliferation, and survival from anti-tumor therapy. Accumulating evidence demonstrates that the EMT is closely related to the aggressive invasiveness and metastasis of epithelial cancer cells [129,130]. In epithelial cancer cells, the EMT process changes the tumor phenotype to a mesenchymal type. Expressions of adhesion molecules that are characteristic of epithelial-type tumors (such as E-cadherin, occludin, and a-catenin) are thus decreased, while expressions of molecules specific to mesenchymal-type tumors (including vimentin, fibronectin, and N-cadherin) are upregulated [131], thus promoting the motility and invasiveness of tumor cells and allowing for the development of tumor cells that are more resistant to apoptosis. As a result, the morphology of tumor cells is changed to that of invasive cells with reduced polarity and a more motile cyto-architecture [132]. The EMT process is activated via a network of EMT-activating transcription factors (EMT-TFs), including TWIST1 [128,133], SNAI1 [134], ZEB1 [135], and SLUG [136]. These transcription factors are regulated by microenvironmental conditions, including the ECM; immune cells, such as macrophages and hypoxia; and miRNAs, including miRNA-200 family members, to acquire the mesenchymal phenotype. Activation of these EMT-TFs is known to correlate positively with CD44 expression. In MCF-7 breast cancer cells, Twist markedly elevates the expression levels of CD44 via activation of the β-catenin and Akt pathways [131]. The overexpression of Slug in MCF-10A and MCF-7 breast cancer cells (CD44-/CD24+) generates a subpopulation of cancer stem cells with a CD44+/CD24+ phenotype showing an enhanced mammosphere-forming ability [136]. On the other hand, CD44 in SW480 colon cancer cells can promote EMT in various cancers by upregulating mesenchymal genes and downregulating epithelial phenotype-related genes [137,138,139]. The overexpression of CD44 downregulates E-cadherin expression; upregulates N-cadherin, α-actin, vimentin, and fibronectin; and disassociates membrane-associated E-cadherin-β-catenin complexes, promoting the translocation of β-catenin into the nucleus and transcriptional activation of genes related to cell invasion and migration [139].

The EMT is also affected by cytokines and hypoxia of the microenvironment surrounding tumor cells. Accumulating evidence suggests that TGF-β is an important cytokine for inducing the EMT and increasing the number of cancer stem cells [140]. The treatment of GBM cells with TGF-β upregulates the expressions of fibronectin and COL5A1 mesenchymal markers, resulting in aggressive tumors showing increased sphere formation and a spindle-shaped morphology, and the effect is reversed with the inhibition of TGF-β [141]. TGF-β receptor type I (RI) is known to contain a CD44-binding site. HA binding to CD44 induces a complex between CD44 and TGF-βRI and stimulates TGF-βRI serine/threonine kinase activity, leading to increased phosphorylation of Smad2/Smad3 and activation of ZEB1, thus causing increased invasiveness [142]. In addition, activated TGF-βRI kinase phosphorylates CD44, resulting in enhanced interactions of CD44 with the cytoskeletal protein ankyrin, in turn promoting HA-CD44 signaling. Luo et al. reported that TGF-β can induce MES transformation via the PDK1/c-Jun pathway by activating N-cadherin, ZEB1, SNAIL, and TWIST1 [143]. TNF-α, which is a common cytokine in the TME, induces mesenchymal transition through the activation of nuclear factor-kappa B (NF-κB) in various types of cancer, including GBM. The treatment of PN-type GSCs with TNF-α causes an MES phenotypic shift with a high expression of CD44 [144]. Iwata et al. reported that the treatment of PN GSCs with TNF-α dramatically increases NF-κB activity, whereas the silencing of NF-κB decreases inducible T-cell costimulator ligand (ICOSLG) expression, which is typically associated with the MES phenotype after TNF-α treatment [145].

Isoforms of CD44 were reported to play a crucial role in causing the EMT in cancer cells [146]. Generally, a switch in CD44 isoform expression from CD44v to CD44s is required to execute the EMT [147]. CD44 alternative splicing is differentially regulated during the EMT [147] so that CD44v can change to CD44s in human immortalized epithelial cells. The CD44s isoform can promote the EMT and its knockdown inhibits the EMT. The re-expression of CD44s fully recovers the phenotype that cannot be obtained from the EMT due to inhibition via CD44 knockdown. Although CD44v is reportedly predominantly expressed in cancer stem-like cells, not only expression patterns and frequency but also the functional significance of CD44s and CD44v are not fully understood. Jijiwa et al. reported that CD44v6 is expressed on GBM stem-like cells and enhances in vitro and in vivo tumor growth, with a link to the upregulated expression of OPN (a ligand of CD44v6) [54]. This result suggests that CD44v6 may promote the generation of GSCs with the epithelial phenotype. Many studies reported CD44v6 as an isoform that promotes the EMT, where its expression is largely restricted to the advanced stages of tumor progression and is more prevalent in metastatic tumors than in non-metastatic ones [51].

How the phenotypic transition of GBM or GBM stem cells plays a role in GBM recurrence has yet to be clarified. A better understanding of the regulation of the functional activities of CD44 in invasion, proliferation, and phenotypic transitions between the invasive type and proliferative type may present a clue to elucidating the molecular mechanisms underlining recurrence in GBM and identifying a therapeutic target for effective treatment against GBM.

## 8. Molecular Mechanism Underlying GBM Recurrence: From the Perspective of the Dual Activity of CD44: Invasion and Proliferation

CD44 participates in multiple cellular processes in cancers, including GBM, as not only an adhesion molecule but also a signal-transducing molecule. In the various cellular processes, invasion and proliferation are two major events in the tumor progression and recurrence of GBM. These two events cannot be executed simultaneously in the same cells, as mentioned above. When tumor recurrence occurs locally at the margin of the resection cavity in GBM, migrated GSCs too far from the original tumor site appear to move to the tumor resection site as GSCs home in on their tumor niche [148,149,150]. Then, to allow for highly migratory GSCs to colonize marginal sites around the resection cavity, a shift in phenotype from highly migratory to less migratory, more proliferative types is necessary. These behaviors of tumor cells are likely to represent the two-way shifts of the PMT and its reversion in the mesenchymal-to-proneural transition (MET). In CD44-regulated signaling pathways, the MAPK and PI3K downstream pathways show mutually exclusive expressions; that is, the former activates the proliferation of tumor cells (corresponding to the epithelial type) and the latter promotes invasion by tumor cells (corresponding to the mesenchymal type), as mentioned earlier [115]. How these signaling pathways are regulated to achieve specific behaviors in tumor cells remains elusive, but a phenotypic transition is likely to play a critical role in displaying the features of each phenotype. A recently proposed definition is that the EMT is a process showing a shift toward the mesenchymal state and includes multiple and dynamic transitional states between the epithelial and mesenchymal phenotypes, as opposed to a process involving a single binary switch [151]. Studies in mouse tumor models and human circulating tumor cells revealed that the EMT is not a binary process during cancer metastasis. In human breast cancer patients, circulating tumor cells show diverse EMT statuses with many varieties of expressions of epithelial and mesenchymal markers [152]. These not-fully EMT cells represent intermediate EMT cells, called partial EMT cells, in which the tumor cells exhibit both mesenchymal and epithelial properties [152]. As described in the section on phenotypic transition, a reversion of the EMT is thought to be essential for the regrowth of invading tumor cells at distant sites. Such EMT/MET switches require the status of partial EMT cells. A full EMT is difficult to reverse in terms of phenotypic features at distant sites in various organs [153]. Further, CD44 isoform switching from the expression of CD44v to CD44s is essential for the EMT [147]. CD44 alternative splicing was differentially regulated during the EMT, resulting in a switch in expression from CD44v to CD44s among human immortalized epithelial mammary cells, as well as breast cancer progression [147]. Zhao et al. reported that the CD44s isoform may play a role in the EMT and CD44v may play a role in tumor engraftment and growth [146].

**Figure 6:**
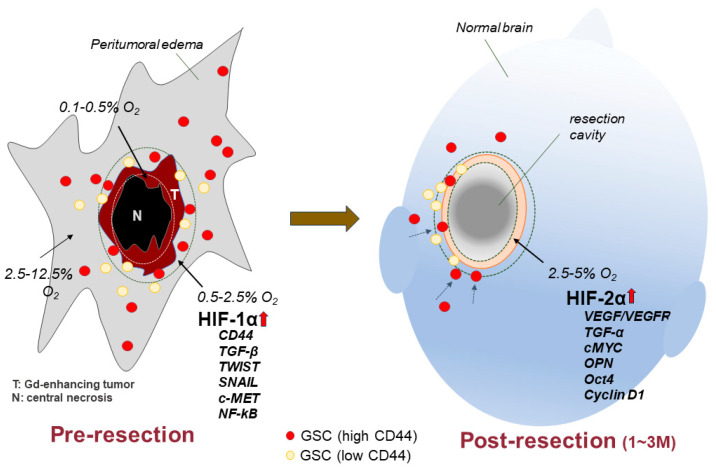
Illustration of hypothetical differential expression of genes related to cell invasion/migration and cell proliferation in glioblastoma according to hypoxic conditions before tumor resection and 1–3 months (in the period of tissue repair) after tumor resection. HIF-1α is upregulated by severe hypoxia of 0.5–2.5% O_2_, corresponding to the tumor rim (tumor border area) before resection. In this area, HIF-1α target genes (including CD44, TGF-β, TWIST, SNAIL, cMET, and NF-kB) are activated, resulting in promotion of cell migration and invasion and enhancement of stemness. HIF-2α is upregulated by moderate hypoxia of 2.5–5% O_2_, corresponding to the marginal area of the resection cavity once tissue injury is repaired and presenting with moderate hypoxia. These cellular processes represent the phenotypic transition of GBM.

Various factors including cytokines, non-tumor cells, and oxygen concentration in the TME can affect the activity of CD44 signaling pathways [154,155]. Among them, hypoxia is a potential factor for executing the phenotypic transition, as the core-to-edge cellular transition of progressive tumor cells is achieved with hypoxia [156]. Consequently, an understanding of the molecular mechanisms underlying GBM recurrence requires clarification of the expression and pathogenesis of various genes and molecules that could be altered under differing levels of hypoxia before and after tumor resection (Figure 6).

Like other solid tumors, the center of the tumor tissue in GBM shows hypoxic features with an oxygen tension of 0.75–4 mmHg (0.1–0.5% O_2_) [157]. In the invasive area at the tumor periphery, oxygen tension may be a little higher than in tumor tissues while remaining hypoxic (0.5–2.5% O_2_) compared with the normal brain (2.5–12.5% O_2_) [157,158]. Under such a hypoxic microenvironment, the expression of various molecules in GSCs may be modified by hypoxia-responding signaling mediated through transcription factors called hypoxia-inducible factors (HIFs) [159]. Among the HIFs, the expression of HIF-1α is upregulated under severe hypoxia (≤1% O_2_), whereas the expression of HIF-2α is upregulated under moderate hypoxia (approximately 5% O_2_) and HIF-2α is expressed predominantly in GSCs [160]. In highly invasive GBM, GSCs with high expression levels of CD44 reside in the peritumoral invasion area when oxygen concentrations show severe-to-moderate hypoxia of 0.5–2.5% O_2_ [100,157]. Such a low oxygen concentration activates HIF-1α, and then the activated HIF-1α upregulates various genes and molecules, including CD44, TGF-β, TWIST, SNAIL, cMET, and NF-κB [161]. Signaling pathways from almost all these genes and molecules are related to the promotion of migration/invasion and the EMT of tumor cells, as shown in the section on signaling pathways. Upregulated CD44 under such severe hypoxia may primarily activate tumor cell migration and invasion rather than tumor cell proliferation [100]. In contrast, the area surrounding the tumor resection cavity in which brain tissues are injured by surgical resection of the tumor shows a severely hypoxic condition in the period immediately after tumor resection. The area including the cavity wall is repaired at least a month after tumor resection, resulting in conditions of moderate hypoxia (2.5–5% O_2_) [162]. In this area, HIF-2α can be predominantly activated, and the activated HIF-2α upregulates different genes and molecules from those activated by HIF-1α. These include VEGF/VEGFR2, cMYC, TGFα, OPN, and Oct4 [70]. Most of these genes and molecules are related to the promotion of proliferation in tumor cells. Under such moderate hypoxia, HIF-2α binds to CD44-ICD and activates various transcription factors, as mentioned above. Kolliopoulos et al. demonstrated that the knockout of CD44 in U251MG cells suppresses cell proliferation, diminishes phosphorylation of CREB, increases the levels of the cell cycle inhibitor p16, and decreases stemness. Similar results were obtained when cells were treated with the γ-secretase inhibitor N-[N-(3,5 difluorophenacetyl-L-alanyl)-(S)-phenylglycine t-butyl ester (DAPT), which inhibits CD44 cleavage, and thus, inhibits the release of ICD from CD44, resulting in the inhibited binding of activated HIF-2α to the released CD44-ICD. This result indicates that transcriptional factors for activating proliferation and stemness in GSCs are dependent on free CD44-ICD [163]. As described in the “CD44-promoted tumor proliferation in GBM (GSCs)” section, OPN can activate γ-secretase by binding to CD44 [15]. We demonstrated that silencing the OPN gene with siRNA increases CD44 expression in GSCs and enhances the CD44-promoted migration and invasion of GSCs [100]. OPN may be a key molecule to promote phenotypic transition in addition to the upregulation of certain transcription factors. The transcription factors NF-κB and c-MYC are reciprocally activated and show the dichotomy between invasion and proliferation according to differences in hypoxic conditions. Under severe hypoxia (<1% O_2_), NF-kB is activated by HIF-1α to enhance the invasive nature of tumor cells at the rim of GBM, whereas c-MYC is upregulated by HIF-2α under moderate hypoxia (2.5–5% O_2_) to promote the proliferation of tumor cells in GBM [164]. In addition, cMET and VEGFR2 are well known to show mutually exclusive expression, as described above. The former is activated by HIF-1α under severe hypoxia and the latter is activated by HIF-2α under moderate hypoxia. These differential expressions of cMET and VEGFR2 can regulate tumor cell growth and invasion according to the binding of ligands HCG and VEGF to the corresponding receptor [67]. These studies indicate that the level of hypoxia may play a critical role in determining the direction of the phenotypic shift induced by CD44. Musah-Eroje, A. et al. described how oxygen gradient regulates phenotypic and genetic changes in GBM and expression of the genes is reversible, showing the plasticity of GBM cells [165]. Understanding the degree of oxygen gradient in GBM will be crucial in clarifying the molecular mechanism underlying GBM recurrence and identifying a target molecule in the treatment for GBM patients.

Hypoxia thus has a strong potential for differentially activating the expression of various genes and molecules for achieving intended cellular processes in tumor cells according to the level of hypoxia. In the process of GBM recurrence, CD44 is expected to play a crucial role in shifting from the mesenchymal type to the epithelial type and vice versa, resulting in the local recurrence of GBM. To confirm whether these mechanisms are operating in the tumor recurrence of GBM, further studies are required. These include clarification of the expressions and functions of related genes and molecules for invasion and proliferation in different levels of hypoxia in much greater detail and whether the same molecular and cellular events can be reproduced using in vivo brain tumor models.

## 9. Conclusions

CD44 is a multi-functional molecule that participates in not only cell adhesion but also signal transduction, and plays a critical role in executing cellular events, such as invasion, migration, proliferation, and tumorigenesis in GBM. CD44 shows functional activity in transducing intra- and intercellular signals in GBM cells. These signaling pathways require CD44 activation to precede signal transduction. The binding of CD44 to HA activates various signaling pathways. Among the pathways used to activate the invasion and proliferation of tumor cells, some pathways show mutually exclusive expression under hypoxic conditions and in different locations in GBM tissues. The PI3K/AKT pathway, which promotes cell invasion and migration, is activated at the tumor rim, whereas the RAS-MARK pathway, which primarily enhances cell proliferation, is activated in the tumor core in the same tumor. NF-κB, which promotes the migration and invasion of tumor cells, is activated by HIF-1α under severe hypoxia. In contrast, cMYC, which enhances cell proliferation, is activated by HIF-2α under moderate hypoxia.

On the other hand, proteolytic cleavage of CD44 can provide specific signaling pathways. The proteolytic action of γ-secretase promotes the cleavage of the transmembrane domain of CD44 and can release CD44-ICD, which translocates to the nucleus and activates various genes as co-transcription factors. Under hypoxic conditions, CD44-ICD binds to HIF-2α and activates HIF-2α expression, which further activates HIF-2α target genes, including cyclin D1, Nanog, Oct4, Sox2, and OPN, resulting in the promotion of tumor proliferation and stemness of GSCs. These results indicate that the dichotomy theory of “Go or Grow” in tumor cells can be executed. At tumor recurrence, GSCs that survive any kind of therapy are required to undergo a phenotypic transition from an invasive type to a proliferative type. Differences in oxygen concentration can allow GSCs to regulate phenotypes to obtain the intended behaviors. These results suggest that CD44 presenting with dual functional activities of invasion and proliferation can play a central role in the generation of GBM recurrence. To elucidate the mechanism underlying the phenotypic transition of CD44 from an invasive to a proliferative type may present a clue to the development of a novel therapeutic method to prevent recurrence in GBM.

## Figures and Tables

**Table 1:** Summary of genes and transcription factors that are activated by CD44-ICD.

Gene	Transcription Factor	Cell Function	O_2_ Condition	References
MMP-9	RUNX2	Invasion	Normoxia	[30]
OPN	(Runt-related transcription factor 2)	Migration	Hypoxia	
		Metastasis		
CD44	CIRE	Invasion	Normoxia	[31]
	(CD44ICD response element)	Proliferation		
		Angiogenesis		
CyclinD1	CREB	Proliferation	Normoxia	[72,73]
PDK1	(cAMP response element-binding protein)	Aerobic glycolysis	Hypoxia	
PFKFB4				
SOX2	HIF-2α	Stemness	Hypoxia	[32,69]
NANOG	(hypoxia-inducible factor-2α)			
OCT4				
c-MYC		Proliferation		

## Data Availability

All data used for analysis are presented in the tables in this article.
